# Synergistic Effects of Liquid Solute Concentration and Cooling Rate on Secondary α_2_-Al Formation in High-Solid-Fraction Rheo-Diecast Al-Si Alloys: An Integrated Experimental and Phase-Field Study

**DOI:** 10.3390/ma19050904

**Published:** 2026-02-27

**Authors:** Song Chen, Wangwang Kuang, Jian Feng, Hongmiao Wang, Daquan Li

**Affiliations:** 1 State Key Laboratory of Nonferrous Structural Materials, China GRINM Group Co., Ltd., Beijing 100088, China; chensong@grinm.com (S.C.); whmhongmiao@163.com (H.W.); 2 GRIMAT Engineering Institute Co., Ltd., Beijing 101407, China; 3 General Research Institute for Nonferrous Metals, Beijing 100088, China; 4 Hongzhiwei Technology (Shanghai) Co., Ltd., Shanghai 200120, China; wwkuang@foxmail.com

**Keywords:** rheo-diecasting, secondary α_2_-Al, phase field, cooling rate, undercooling

## Abstract

The synergistic effects of solute concentration and cooling rate on the evolution of secondary α_2_-Al during high-solid-fraction rheo-diecasting of Al-xSi (x = 1, 4, 7 wt.%) alloys was studied. Combined gradient-cooling experiments (100 vs. 10 K/s) and phase-field simulations show that the population and morphology of secondary α_2_-Al are co-governed by initial Si content and cooling rate. Higher cooling rates promote finer, more uniform secondary α_2_-Al in Al-1Si and Al-4Si, while lower cooling rates cause coarsening and coalescence. In addition, the formation of α_2_-Al is severely suppressed in Al-7Si. Crucially, a lower initial solute concentration significantly amplifies cooling rate-induced solute enrichment, quantitatively evidenced by the final liquid concentration difference (Al-1Si: 0.83 wt.% > Al-4Si: 0.29 wt.% > Al-7Si: 0.13 wt.%). This enrichment governs the dynamic competition between constitutional and thermal undercooling, contributing a substantially greater driving force for early-stage nucleation in Al-1Si compared to Al-7Si. As solidification progresses in all three systems, the enrichment of the residual liquid narrows the solidification interval, thereby progressively elevating the role of thermal undercooling.

## 1. Introduction

Semi-solid rheo-diecasting is an advanced manufacturing technology capable of high-efficiency, near-net-shape forming [1]. By eliminating the secondary reheating step required in traditional thixo-forming, this process offers advantages such as a shortened production cycle, reduced energy consumption, laminar filling, low solidification shrinkage, and high product density with low porosity [2,3]. Consequently, the formed components exhibit mechanical properties comparable to forged parts, making the process particularly suitable for complex thin-walled components with rigorous requirements for lightweight design and reliability in automotive and telecommunication sectors [4]. During rheo-diecasting, the slurry prepared in the first stage is transferred into the mold cavity, and the residual liquid undergoes rapid cooling and secondary solidification. The secondary solidification microstructure, especially the secondary α_2_-Al phase, significantly influences the mechanical properties and hot-cracking tendency of final components. Accordingly, a deep investigation of the nucleation and evolution mechanisms of secondary α_2_-Al, as well as their intrinsic relationship with process conditions, is of great importance for optimizing the rheo-diecasting process and improving component performance.

Previous studies have noted the presence of secondary α_2_-Al and have experimentally conducted preliminary explorations into its formation, nucleation, and growth. Ji et al. [5] investigated the effect of different slurry preparation processes on the secondary solidification microstructure in Sn-15Pb alloys, shifting the research focus from the growth behavior of primary α_1_-Al associated with slurry preparation to the subsequent secondary solidification stage. Liu et al. [6] proposed that lowering the pouring temperature during slurry preparation increases undercooling, thereby promoting the explosive nucleation of secondary α_2_-Al. Regarding the microstructure evolution characteristics and morphology of secondary α_2_-Al, Hitchcock et al. [7,8] systematically elucidated the solidification path of rheo-diecasting in A357 alloy. They noted that high shear rates lead to highly uniform temperature and solute fields within the residual liquid, prompting the simultaneous nucleation and growth of secondary α_2_-Al into fine, spherical particles using the Mullins–Sekerka stability theory. Payandeh et al. [9] revealed the solute concentration differences between primary and secondary α_2_-Al. It is concluded that the solidification sequence and solute enrichment in the residual liquid lead to a reduction in undercooling. Li et al. [10,11] further characterized the morphology and distribution of secondary α_2_-Al under different cooling conditions, proposing that its evolution undergoes three stages: stable growth, instability, and coalescence. Through solute analysis, they confirmed significant enrichment of Si and Mg within the secondary α_2_-Al.

Despite these significant advancements, several critical mechanistic gaps persist. First, current analyses remain largely dependent on static post-solidification microstructure analysis and theoretical inference, lacking real-time quantitative simulations capable of directly and dynamically tracking nucleation and growth of secondary α_2_-Al. Furthermore, investigations have largely been confined to limited alloy compositions, which limits quantitative insight into the synergistic effects of initial solute concentration and cooling rate, especially in controlling secondary α_2_-Al evolution under high-solid-fraction conditions. Consequently, two key quantitative aspects remain unclear. First, the synergistic effects of solute concentration and cooling rate on secondary α_2_-Al formation lack a quantitative description, particularly regarding their joint regulation of solute enrichment and the nucleation driving force. Second, the dynamic competition between thermal and constitutional undercooling during rheo-diecasting has not been elucidated. To address these gaps, this study investigates, through an integrated experimental and simulation approach, the synergistic effects of initial solute concentration and cooling rate on the nucleation behavior, spatial distribution, and final morphology of the secondary α_2_-Al phase, as well as the dynamic evolution of the relative contributions from thermal and constitutional undercooling during the rheo-diecasting process. The revealed mechanism offers a direct theoretical basis and processing guidelines for understanding and predicting the nucleation and evolution of secondary α_2_-Al in Al-Si alloys with different compositions.

## 2. Experimental Procedure

### 2.1. Model Materials Choice

Three hypoeutectic Al-Si alloys, that is, Al-1Si, Al-4Si, and Al-7Si (all in wt.%), were employed in this study, which were prepared by melting high-purity Al (99.7 wt.%) with the Al-20Si master alloy. The constituents were proportioned to achieve the desired silicon concentrations. After degassing with a rare-earth agent at 720–740 °C, the high-purity melt was cast into a special steel mold for composition analysis. The actual chemical compositions were determined by inductively coupled plasma atomic emission spectrum (ICP-AES) apparatus (SPECTRO ARCOS, SPECTRO, Kleve, Germany), and the results are shown in Table 1.

**Figure 1:**
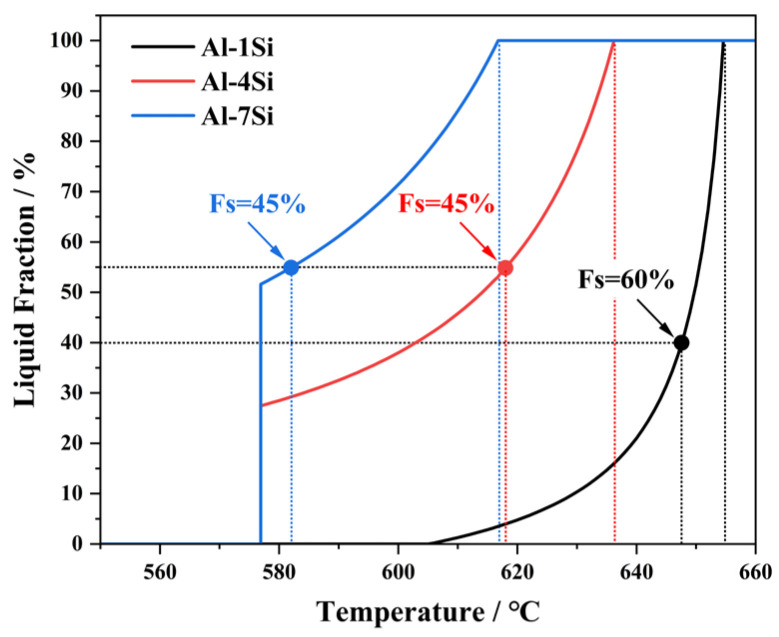
Solid fraction (Fs)-temperature (T) profile of Al-1/4/7Si alloy simulated with Pandat software and each targeted slurry condition.

This alloy series was designed to examine how the initial Si content affects secondary solidification under high-solid-fraction conditions. And the solidification path was simulated by Pandat 2023 software (CompuTherm LLC, Marlborough, MA, USA, 2023 version). As illustrated in Figure 1, the equilibrium phase diagram reveals that increasing the Si content from 1 wt.% to 7 wt.% significantly alters the solidification pathway: the alloy transitions from a single-phase solidification without eutectic reaction in Al-1Si. The maximum equilibrium solid fraction attainable before the eutectic temperature is reached is approximately 72% for Al-4Si and 50% for Al-7Si [12]. To systematically compare the influence of solute concentration and high-solid-fraction on secondary solidification, the target Stage I solid fractions were selected as follows: 60% for Al-1Si, and 45% for both Al-4Si and Al-7Si. Especially, Al-1Si alloy was designed to operate under a higher-solid-fraction (Fs = 60%), low-solute extreme condition. This setup enables a clear investigation of how solute level couples with cooling rate to control the nucleation and evolution of secondary α_2_-Al.

The selected compositions (1, 4, and 7 wt.% Si) strategically cover the industrially relevant hypoeutectic range for semi-solid processing. Specifically, the Al-7Si composition corresponds directly to the widely used rheo-diecast alloy A356/A357 (~7% Si), a primary target for semi-solid forming. The Al-4Si model alloy represents an intermediate solute level, allowing the continuous variation of solute effects to be revealed. Moreover, the Al-1Si alloy serves to probe the limiting behavior under low-solute conditions. Additionally, its composition aligns with the silicon content of commercial wrought alloys such as 6013 (~1.0% Si), which are of growing interest for net-shape rheo-diecasting applications (replacing forging with casting) that require a balance of strength and anodizability.

### 2.2. Solidification Process

**Figure 2:**
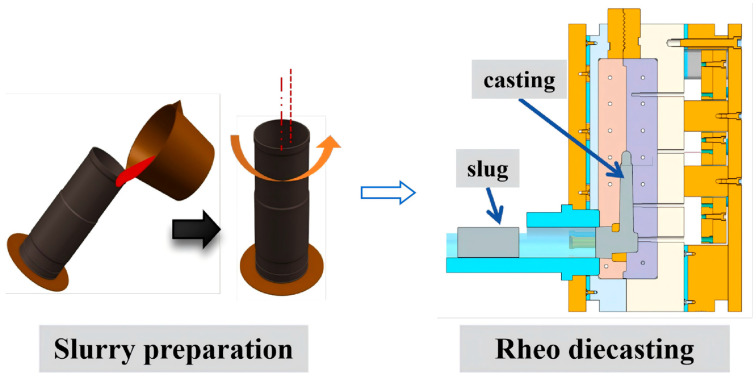
Schematic diagram of high-solid-fraction slurry preparation (Stage I) and rheo-diecasting process (Stage II).

High-solid-fraction semi-solid slurries were fabricated using the swirling enthalpy equilibration device (SEED) method [13,14,15]. The relationship between the solid fraction (Fs) and temperature for the alloys was derived from their equilibrium phase diagrams, as illustrated in Figure 1. In the first stage, the molten alloy was poured into an open crucible (Figure 2), where the thermal evolution was recorded in situ by a φ2 mm K-type thermocouple. The average cooling rate was measured to be approximately 0.1 K/s, which approaches near-equilibrium solidification conditions [16,17,18]. Based on the measured cooling curves and the corresponding phase diagrams, the solid fraction of the high-solid-fraction slurry could be controlled by adjusting the swirling time. Contemplating the variation in liquidus temperature due to different silicon contents, the pouring temperatures were set to 665 ± 2 °C, 650 ± 2 °C, and 630 ± 2 °C for the 1Si, 4Si, and 7Si alloys, respectively. By optimizing the swirling time, slurries with average solid fractions of 60% (1Si) and 45% (4Si and 7Si) were achieved.

In the second stage, an experimental mold designed to induce a gradient-cooling rate was employed for the rheo-diecasting process. Due to the experimental challenges associated with in situ measurement of the rapid cooling rates within the die cavity, finite element method (FEM) simulations were conducted using ProCAST 2009 software (ESI Group, Paris, France, 2009 version). These simulations provided a comprehensive mapping of the macroscopic temperature field evolution. And the local cooling rates were quantified by calculating the first derivative of the temperature–time profiles (dT/dt) [17,18,19]. Consequently, two representative locations were identified for microstructure analysis: Position A near the mold surface, corresponding to a high cooling rate of 100 K/s, and Position B in thicker sections, corresponding to a moderate cooling rate of 10 K/s. Thus, the selected cooling rates of 10 K/s and 100 K/s were strategically chosen to encompass the practically relevant range in rheo-diecasting, allowing us to systematically investigate the influence of an order-of-magnitude variation in solidification kinetics on the nucleation and growth of secondary α_2_-Al. All experiments were repeated under the same equipment and process parameters to ensure process consistency. The cooling curves and solid fraction control were verified through multiple experiments.

### 2.3. Microstructure Characterization

**Figure 3:**
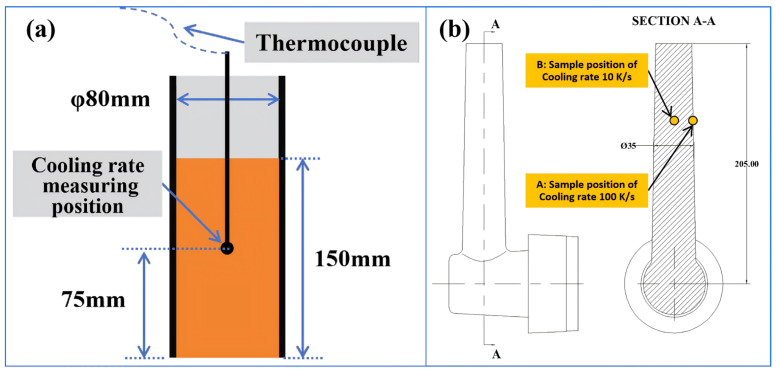
Experimental cooling curve monitoring for high-solid-fraction slurry preparation (Stage I) and sampling points in the 3D gating system of different cooling rate positions simulated by ProCAST: (**a**) the cooling rate measuring position of stage I, (**b**) the casting and sample positions of stage II.

The locations for metallographic (OM) and scanning electron microscopic (SEM) examination are shown in Figure 3b. For each alloy/process combination, at least three independent samples were prepared and analyzed to confirm the stability and statistical significance of the microstructural features. Specimens were ground with 400#, 1000#, and 2000# SiC abrasive papers, after which the samples were mechanically polished and etched with Keller’s reagent (2 mL HF, 3 mL HCl, 5 mL HNO_3_, 190 mL H_2_O). An Axiovert 200 MAT light microscope (Carl Zeiss, Oberkochen, Germany) was employed for microstructural inspection. Further microstructural and compositional analyses were conducted using a ZEISS Sigma 300 scanning electron microscope (Carl Zeiss, Oberkochen, Germany) fitted with an Oxford Ultim Max65 energy-dispersive X-ray spectrometer (EDS). The SEM was operated at 20 kV with working distances between 10 and 15 mm. In addition, an electron probe micro-analyzer (EPMA, JXA-iHP200F, JEOL, Tokyo, Japan) was utilized for high-spatial-resolution chemical analysis and detailed morphological observation. The EPMA was run at 30 kV with an 11 mm working distance, employing wavelength-dispersive spectroscopy (WDS) to acquire quantitative compositional data from selected micro-areas.

## 3. Phase-Field Simulation

Phase-field modeling employs continuous order parameter fields to represent phase distributions, thereby avoiding the need for explicit interface tracking. This approach is particularly effective for simulating complex morphological evolution during solidification [20,21,22]. Within the framework, two types of field variables are defined: a non-conserved field, which describes the local phase state (often interpreted as the phase volume fraction) and evolves according to the Allen–Cahn equation [23]; and the composition evolution equation is often referred to as the Cahn–Hilliard equation [24]. These coupled equations provide a diffuse-interface description that naturally captures interfacial dynamics and solute redistribution during solidification. In the current work, to effectively capture the spontaneous nucleation of α_2_-Al phase during continuous cooling, based on the classical Karma–Rappel (K-R) model [25,26,27], we enhanced the phase-field model with two phases (α-Al phase and liquid phase) by incorporating a spontaneous nucleation module and integrating time-dependent thermodynamic parameters. Input parameters for the phase-field model are based on experimental data or well-established thermodynamic databases. The simulations are deterministic and reproducible under given initial conditions. Key parameters are listed in Table 2. The details are provided below.

### 3.1. Phase-Field Equations

Solidification dynamics are driven by solute concentration gradients across the solid–liquid interface, which dictate its migration. To simulate this process, the local solid phase fraction is represented by an order parameter ϕ. A value of ϕ = 1 corresponds to the pure solid state, whereas ϕ = 0 represents the liquid state. Across the diffuse interface, ϕ exhibits a continuous variation from 0 to 1. The control equation describing the behavior of ϕ can be written as [19,28,29,30]:

(1) $$\tau \left[1+\left(1-k\right)u\right]a_{s}^{2}\left(\overset{\cdot \to }{n}\right)\partial_{t}\phi =W^{2}\nabla \cdot \left[a_{s}^{2}\left(\overset{\cdot \to }{n}\right)\nabla \phi \right]+W^{2}\partial_{x}\left[{\left|\nabla \phi \right|}^{2}a_{s}\left(\overset{\cdot \to }{n}\right)\frac{\partial a_{s}\left(\overset{\cdot \to }{n}\right)}{\partial \left(\partial_{x}\phi \right)}\right]\;+W^{2}\partial_{y}\left[{\left|\nabla \phi \right|}^{2}a_{s}\left(\overset{\cdot \to }{n}\right)\frac{\partial a_{s}\left(\overset{\cdot \to }{n}\right)}{\partial \left(\partial_{y}\phi \right)}\right]+W^{2}\partial_{z}\left[{\left|\nabla \phi \right|}^{2}a_{s}\left(\overset{\cdot \to }{n}\right)\frac{\partial a_{s}\left(\overset{\cdot \to }{n}\right)}{\partial \left(\partial_{z}\phi \right)}\right]\;-2\phi \left(1-\phi \right)\left(1-2\phi \right)-8\lambda u\phi^{2}{\left(1-\phi \right)}^{2}$$

For the solute concentration c (mole fraction), its governing equation is [27]:

(2) $$\partial_{t}c=\nabla \cdot \left\{D_{\phi }\left[\nabla c+c\frac{1-k}{1-\left(1-k\right)\phi }\nabla \phi \right]-\frac{Wc}{\sqrt{8}}\frac{\left(1-k\right)}{1-\left(1-k\right)\phi }\partial_{t}\phi \frac{\nabla \phi }{\left|\nabla \phi \right|}\right\}$$

*u* represents the solid supersaturation, which is expressed as:

(3) $$u=\frac{\frac{c/c_{l}^{eq}}{1-\left(1-k\right)\phi }-1}{1-k}$$

where $c_{l}^{eq}$ is the equilibrium liquid-phase concentration, and the equilibrium solute partition coefficient k(T) and the equilibrium liquid composition $c_{l}^{eq}(T)$ are no longer constants but functions that evolve in real time with temperature, which are obtained from phase diagram calculations in the current work. Consequently, the supersaturation *u* and, thus, the entire phase-field kinetics are dynamically coupled to CALPHAD through these two core thermodynamic parameters [31,32].

Where

(4) $$D_{\phi }={\;D}_{l}\left(1-\phi \right)+D_{s}\phi$$

is the effective diffusion coefficient with $D_{l}$ is the liquid-phase diffusion coefficient, $D_{s}$ is the solid-phase diffusion coefficient, k is the equilibrium solute partition coefficient of the solid phase. For more details about the model, the readers can refer to Ref. [19].

### 3.2. Implementation of Explicit Nucleation Model

During the rheo-diecasting stage (Stage II), the semi-solid slurry comprises primary α_1_-Al phases dispersed in a liquid matrix. As solidification proceeds, secondary α_2_-Al grains nucleate independently and grow within the confined residual liquid channels. To accurately characterize the kinetics of this secondary nucleation event, the explicit nucleation algorithm developed by Simmons et al. [33,34] was implemented in the present simulation.

This algorithm bridges phenomenological nucleation theory and numerical modeling by translating the continuous deterministic framework of classical nucleation theory into a discrete stochastic process, integrated within a phase-field time-stepping scheme. Within this explicit approach, potential nucleation sites are generated stochastically throughout the domain, yet the actual phase transformation is only triggered when the local undercooling exceeds a critical threshold. Consequently, microstructural evolution involves both the growth of pre-existing grains and the stochastic emergence of new nuclei within the solute-enriched liquid as the thermal driving force increases.

For a given spatial location, the local nucleation rate (*J*) is expressed as:

(5) $$J=Z\beta^{*}exp\left(\frac{-\Delta G^{*}}{k_{B}T}\right)exp\left(\frac{-\tau_{N}}{t}\right)$$

Therein, *Z* is the Zeldovich factor, β* is the frequency factor for the growth of the critical nucleus into a supercritical nucleus, ΔG* is the activation energy of the critical nucleus related to local composition and temperature, *τ_N_* is the nucleation incubation time, *k_B_* is the Boltzmann constant, and *T* is the temperature.

Within the explicit nucleation algorithm employed in the phase-field simulations, the continuous nucleation rate is transformed into discrete stochastic nucleation events via a Poisson statistical model. The core of this approach lies in describing the probability of such rare events occurring within a specified time interval and spatial volume. Assuming nucleation events are random and independent, their statistics follow a Poisson distribution. Specifically, in the phase-field simulation domain, for any individual grid cell (x, y) within the remaining liquid region and within a time step Δ*t*, the expected average number of nucleation events *λ* is given by:

(6) λ=JΔt

In the phase-field simulations, the local nucleation rate is denoted by *J*. Under the assumption that nucleation events are stochastic and independent, their occurrence within a given spatiotemporal interval follows a Poisson distribution: the probability of *k* nucleation events is given by $P(k)\;=\;\frac{\lambda^{k}e^{-\lambda }}{k!}$, where *λ* represents the expected average number of nucleation events in that interval. Consequently, the probability of no nucleation occurring is $P\left(0\right)\;=\;e^{-\lambda }$. From this, the probability of at least one nucleation event within a time step Δ*t* is derived as:

(7) $$P=1-exp\left(-J\Delta t\right)$$

During the simulation, a random number R uniformly distributed between 0 and 1 is generated at each potential nucleation site. If R < P, a nucleation event is triggered at that location by setting the corresponding order parameter ϕ to 1.

It should be noted that the complete expression for the nucleation rate is relatively complex and involves several parameters that are difficult to quantify precisely, which poses practical challenges for direct implementation in phase-field simulations. Therefore, based on its mathematical form, we adopt a simplified version as follows:

(8) $$J\;=\;k_{1}exp\left(\frac{-k_{2}}{u}\right)$$

where $k_{1}$ and $k_{2}$ are adjustable constants.

The simplified expression (Equation (8)) preserves the physical essence of the original nucleation rate equation (Equation (5)) while consolidating several difficult-to-determine parameters into two tunable coefficients. The activation energy for nucleation is $\Delta G^{*}$ = *πγ*^2^/ΔG, *γ* is the interfacial energy, and ΔG is the free energy change per unit area of the transformation, and ΔG ≈ *const* × *u*, where *u* is the supersaturation and *k*_1_ = *Z*$\beta^{*}$ and *k*_2_ =* πγ*^2^/(*const* × *kT*) [33]. The same values of *k*_1_ and *k*_2_ used in this study are taken from our prior validated work [19]. This approach preserves the predictive reliability of the model while significantly enhancing its practicality and computational efficiency in phase-field simulations.

### 3.3. Coupling of Thermodynamic Databases

**Figure 4:**
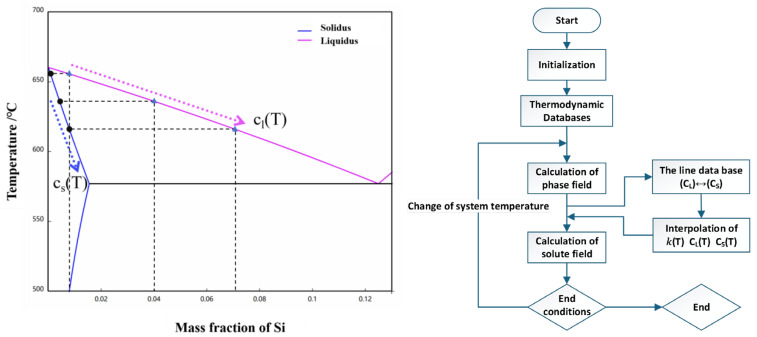
The equilibrium phase diagram of the Al-Si alloy and the flowchart of phase-field simulation coupled with phase diagram data.

During rheo-diecasting, solidification proceeds under continuous cooling and non-equilibrium conditions, leading to a continuous evolution of the liquid composition $c_{l}(T)$ with decreasing temperature. Consequently, the corresponding liquidus temperature $T_{Liquid}[c_{l}^{\mathrm{eq}}(T)]$ also varies dynamically, which is directly derived from Al-Si equilibrium phase diagram data. To accurately capture microstructural evolution and secondary α_2_-Al nucleation under such transient conditions, at each simulation time step, the current system temperature T is passed to the thermodynamic database to retrieve and update the values of *k*(*T*) and $c_{l}^{\mathrm{eq}}$(T). By dynamically interfacing with the thermodynamic database during simulation, key temperature-dependent parameters are retrieved on the fly, including the equilibrium solute partition coefficient k(T), the equilibrium liquid and solid compositions $c_{l}^{\mathrm{eq}}(T)$ and $c_{s}^{\mathrm{eq}}(T)$, as well as the liquidus temperature $T_{Liquid}[c_{l}^{\mathrm{eq}}(T)]$ corresponding to the current composition [31,32]. The implementation scheme for the real-time integration of thermodynamic parameters into the phase-field simulation is illustrated in Figure 4.

For the Al-Si system, the thermodynamic database referenced in Ref. [12] was employed. Due to unavailable temperature-dependent diffusion coefficients, these parameters are treated as constants. All simulation parameters are listed in Table 2 [19].

In this study, the macroscopic temperature field evolution during the solidification stage of rheo-diecasting (Stage II) was obtained through finite element numerical simulation. The resulting cooling rate under non-equilibrium solidification conditions was introduced as a multiscale input boundary condition for the phase-field simulations. Coupled with a dynamic phase diagram database, this approach enables real-time updating of temperature-dependent thermodynamic parameters, such as the solute field. Consequently, a multiscale thermo-kinetic simulation of the full cooling trajectory from slurry preparation to rheo-diecasting solidification was achieved, capturing the complete microstructure evolution. Detailed results and corresponding analyses are presented in the following sections.

## 4. Results

### 4.1. Rheo-Diecasting Experiment Results

**Figure 5:**
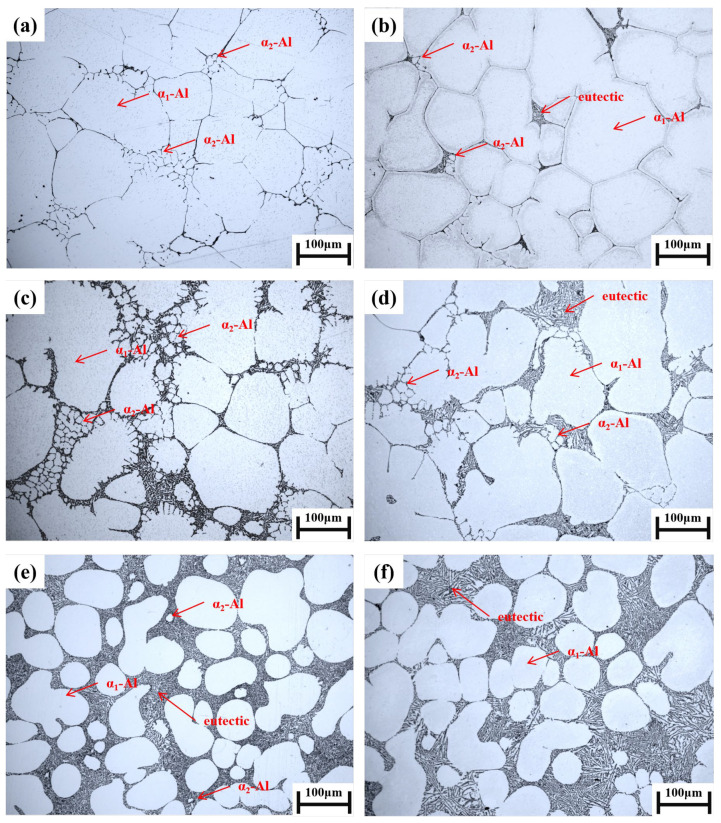
Microstructures of rheo-diecasting Al-xSi alloys under varied solid fractions and cooling rates: (**a**) Al-1Si, Fs = 60% (Stage I), and 100 K/s (Stage II); (**b**) Al-1Si, Fs = 60%, and 10 K/s; (**c**) Al-4Si, Fs = 45%, and 100 K/s; (**d**) Al-4Si, Fs = 45%, and 10 K/s; (**e**) Al-7Si, Fs = 45%, and 100 K/s; (**f**) Al-7Si, Fs = 45%, and 10 K/s.

The microstructure of Al-1Si, Al-4Si, and Al-7Si alloys with two cooling rates (100 K/s and 10 K/s) is shown in Figure 5. In the Al-1Si alloy (Figure 5a,b), the residual liquid region is surrounded by the primary α_1_-Al. The secondary α_2_-Al appears spherical or short rod-like, distributed near the grain boundaries. As the cooling rate decreases from 100 K/s to 10 K/s, the size of the α_2_-Al slightly coarsens, and the number significantly decreases. At 100 K/s, the secondary α_2_-Al is uniformly distributed around the grain boundaries of primary α_1_-Al, with some phases appearing as short-rod morphology.

With the Al-4Si alloy (Figure 5c,d), the residual liquid region expands noticeably, and the number of secondary α_2_-Al is significantly greater than that in the Al-7Si alloy. These secondary phases, appearing spherical or interconnected in a network, are distributed between the primary α_1_-Al grain boundaries. Under the high cooling rate of 100 K/s, the secondary α_2_-Al near grain boundaries exhibits a spherical morphology within the residual liquid region. Those adjacent to primary α_1_-Al boundaries tend to grow attached to the primary phase, resulting in some primary α_1_-Al grains appearing globular with toe-like protrusions. At 10 K/s, the size of secondary α_2_-Al significantly increases, and the tendency for coalescence with primary α_1_-Al at grain boundaries becomes more pronounced.

For the Al-7Si alloy (Figure 5e,f), the formation of secondary α_2_-Al is strongly suppressed because the solid fraction of the slurry in Stage I already approaches the equilibrium solid fraction corresponding to the eutectic reaction. Under the high cooling rate of 100 K/s (Figure 5e), a limited number of isolated spherical secondary α_2_-Al particles are observed within the residual liquid region. At 10 K/s (Figure 5f), the discrete secondary α_2_-Al phases are hardly discernible.

Comparing the microstructures of the three alloys under the high cooling rate of 100 K/s (Figure 5a,c,e) reveals that secondary α_2_-Al is scarcely present in the residual liquid region of the Al-7Si alloy, whereas noticeable amounts of secondary α_2_-Al exist along the grain boundaries in both Al-1Si and Al-4Si alloys. It is noteworthy that even under a high solid fraction of 60% and a low cooling rate of 10 K/s (Figure 5b), secondary α_2_-Al can still precipitate from the remaining liquid in the Al-1Si alloy, indicating the possibility of secondary α_2_-Al formation at a high solid fraction. Moreover, the Al-4Si alloy, with a solid fraction of 45% in the first stage, can still form a considerable amount of secondary α_2_-Al in the remaining liquid during solidification (Figure 5d), and these grow synergistically with the primary α_1_-Al. This marked contrast with the Al-7Si alloy (Figure 5f) indicates that the nucleation and growth of secondary α_2_-Al are governed not only by the cooling rate but also, decisively, by the solute concentration in the remaining liquid phase.

**Figure 6:**
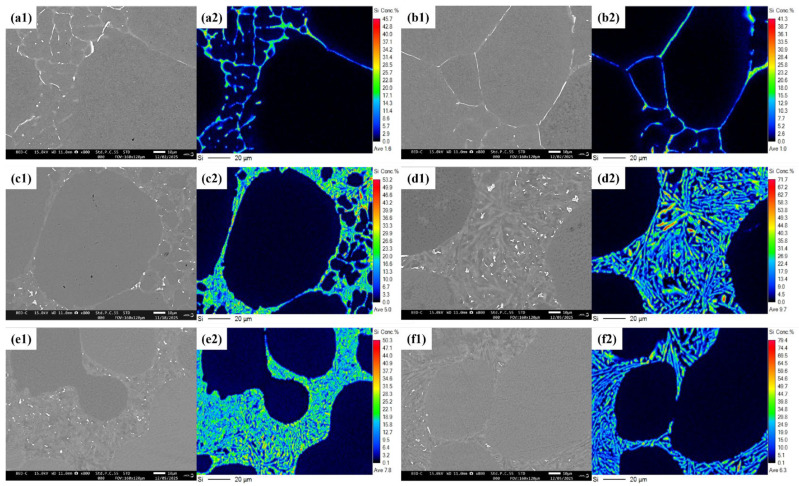
SEM microstructures and EPMA Si-mapping of semi-solid rheo-diecasting Al-xSi alloys under varied solid fractions and cooling rates: (**a**,**b**) Al-1Si (Stage I: Fs = 60%), (**c**,**d**) Al-4Si, and (**e**,**f**) Al-7Si (Stage I: Fs = 45%). Stage II cooling rates are 100 K/s (**a**,**c**,**e**) and 10 K/s (**b**,**d**,**f**). (**a1**,**b1**,**c1**) SEM microstructure, (**a2**,**b2**,**c2**) EPMA Si mapping.

To further identify the fine-phase composition within the residual liquid region, scanning electron microscopy (SEM) observations and electron probe microanalysis (EPMA) for Si elemental mapping were performed on samples with different compositions (Al-1Si, Al-4Si, Al-7Si) under cooling rates of 100 K/s and 10 K/s, which were shown in Figure 6. In the Al-1Si alloy, secondary α_2_-Al is distributed between primary α_1_-Al grains, with some coalescing near grain boundaries. As the grain boundary region gradually narrows during the secondary solidification process, the EPMA results show that the secondary α_2_-Al exhibits a mesh-like distribution in the residual liquid region, where secondary phases merge with the primary phase, leading to toe-like protrusions along certain primary grains. Even at the lower cooling rate of 10 K/s, the secondary α_2_-Al is still generated, and its size is coarser compared to that at 100 K/s.

As the Si content increases to 4.0%, the proportion of the residual liquid region significantly increases. At 100 K/s, nearly spherical secondary α_2_-Al phases are distributed within the residual liquid region, with coalescence observed among them. At 10 K/s, very fine secondary α_2_-Al may be present, but it is difficult to distinguish due to the small contrast difference with the eutectic morphology. Further increasing the Si content to 7.0% severely suppressed the formation of secondary α_2_-Al. This is attributed to the higher solid fraction (45%) of the slurry in Stage I, which is close to the eutectic point, and the consequent near-eutectic composition of the remaining liquid. At 100 K/s, only a few isolated secondary α_2_-Al particles are sporadically observed within the residual liquid region. At the lower cooling rate of 10 K/s, secondary α_2_-Al cannot be clearly distinguished in terms of either morphology or composition.

### 4.2. Phase-Field Simulation Results

Although experimental techniques can characterize the final microstructure morphology and distribution, they are unable to capture the nucleation events of secondary α_2_-Al in real time, track the transient evolution of the solute field during solidification, or quantitatively resolve the dynamic competition between thermal and constitutional undercooling. Phase-field simulation offers an indispensable approach in this context: based on realistic thermodynamic data, it can dynamically reconstruct the solidification pathway at the mesoscopic scale, resolve the spatiotemporal distribution of the solute field in real time (including the continuous evolution of the solute concentration in the remaining liquid), visually reveal the coupling between the nucleation and evolution of secondary α_2_-Al and solute redistribution.

The simulation parameters aligned with the experimental conditions: the Al-1Si alloy, with a Stage I target solid fraction of 60%, was simulated at Stage II cooling rates of 10 K/s and 100 K/s. Both the Al-4Si and Al-7Si alloys, with a final solid fraction of 45% in Stage I, were simulated under the same Stage II cooling rates for comparison.

**Figure 7:**
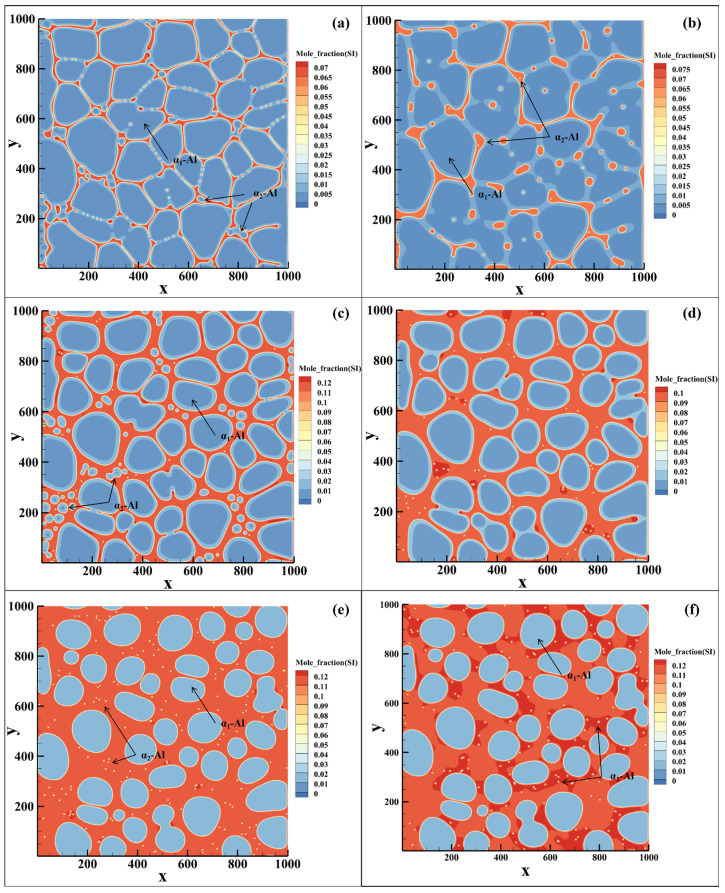
Phase-field simulated morphology of secondary α_2_-Al under varied Si contents and cooling rates (Stage II): (**a**) Al-1Si (Stage I: Fs = 60%)-100 K/s, (**b**) Al-1Si (Stage I: Fs = 60%)-10 K/s, (**c**) Al-4Si (Stage I: Fs = 60%)-100 K/s, (**d**) Al-4Si (Stage I: Fs = 60%)-10 K/s, (**e**) Al-7Si (Stage I: Fs = 45%)-100K/s and (**f**) Al-7Si (Stage I: Fs = 45%)-10K/s.

The phase-field simulation results are shown in Figure 7. Figure 7a,b show the microstructure of the Al-1Si alloy upon reaching the solidus temperature at cooling rates of 100 K/s and 10 K/s, respectively. Under the high cooling rate, nearly spherical secondary α_2_-Al phases form within the residual liquid, with some coalescing with primary α_1_-Al to produce toe-like protrusions along primary grain boundaries. At the lower cooling rate, microstructure evolution is dominated by the coalescence of primary α_1_-Al, with only a limited number of fine secondary α_2_-Al particles forming.

The corresponding results for the Al-4Si alloy are presented in Figure 7c,d. At 100 K/s, a substantial number of spherical secondary α_2_-Al particles nucleate in the residual liquid, some evolving into short-rod morphology through growth and coalescence. At 10 K/s, while nucleation still occurs, the subsequent growth is limited by an insufficient driving force and the potential re-dissolution of unstable nuclei caused by latent heat release.

For the Al-7Si alloy, the growth of secondary α_2_-Al was severely limited because the solute concentration in the residual liquid approaches the eutectic composition (approximately 12.6 wt.% Si). Secondary α_2_-Al particles are uniformly distributed within the residual liquid regions. The subsequent eutectic reaction and associated latent heat release may promote the re-dissolution of unstable nuclei, which accounts for the experimental difficulty in distinguishing secondary α_2_-Al within the residual liquid region.

**Figure 8:**
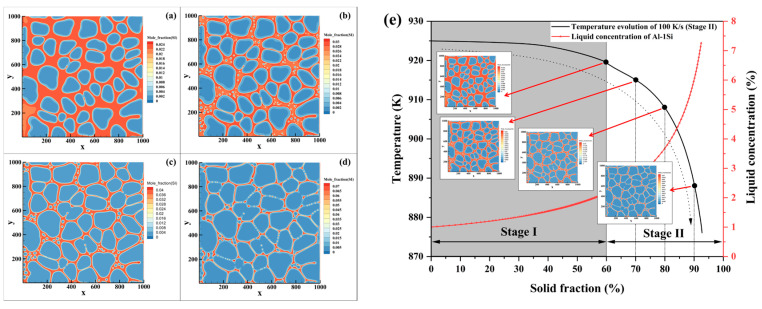
Phase-field simulation of microstructure evolution during continuous cooling rheo-diecasting of an Al-1Si alloy, after switching to 100 K/s at a Stage I solid fraction of 60%, shown at solid fractions of (**a**) 60%, (**b**) 70%, (**c**) 80%, (**d**) 90% and (**e**) its evolution path.

**Figure 9:**
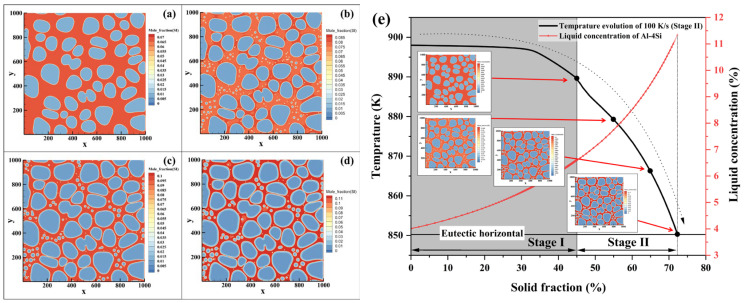
Phase-field simulation of microstructure evolution during continuous cooling rheo-diecasting of an Al-4Si alloy, after switching to 100 K/s at a Stage I solid fraction of 45%, shown at solid fractions of (**a**) 45%, (**b**) 55%, (**c**) 65%, (**d**) 72% and (**e**) its evolution path.

**Figure 10:**
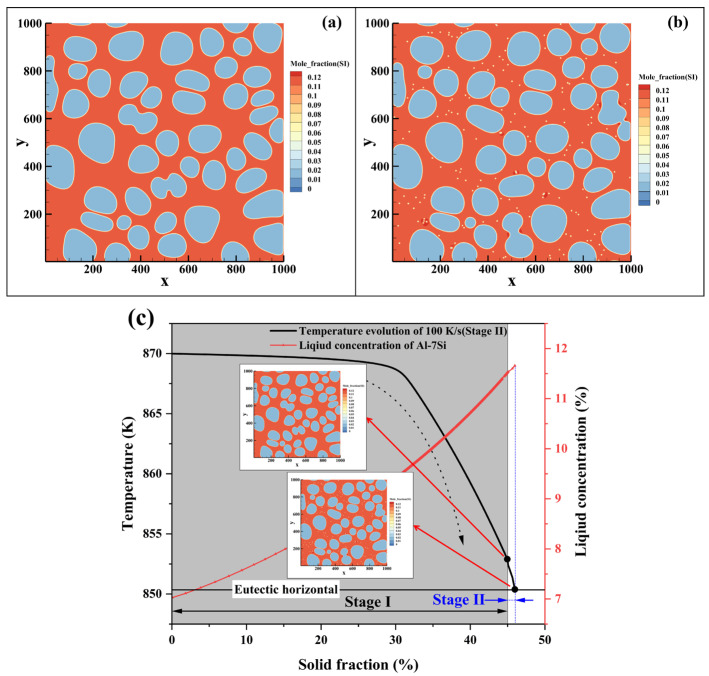
Phase-field simulation of microstructure evolution during continuous cooling rheo-diecasting of an Al-7Si alloy, after switching to 100 K/s at a Stage I solid fraction of 45%, shown at solid fractions of (**a**) 45%, (**b**) 47%, and (**c**) its evolution path.

To elucidate the synergistic control of cooling rate and solute concentration in the residual liquid on the nucleation and growth of secondary α_2_-Al, phase-field simulations were systematically performed to analyze the microstructure evolution during rheo-diecasting solidification (Stage II) under a high cooling rate of 100 K/s for Al-1/4/7Si alloys. The solute field simulation results for the three alloys are presented in Figure 8, Figure 9 and Figure 10, respectively, where the spatiotemporal distributions of solute are displayed to clarify the influence of liquid composition evolution on the formation of secondary α_2_-Al.

In the Al-1Si alloy (Fs = 60%), the solute concentration in the residual liquid is only about 2.17 wt.% Si after switching to the high cooling rate (Figure 8). A large number of spherical secondary α_2_-Al particles form in the liquid. As solidification proceeds, some of the smaller or less stable particles dissolve due to compositional fluctuations, while stable nuclei continue to grow spherically. Owing to the wide solidification temperature range of this alloy, adjacent secondary α_2_-Al particles exhibit a tendency to coalesce during growth. Simultaneously, cooperative growth between secondary and primary α-Al leads to continuous solute enrichment in the remaining liquid. This enrichment, together with the gradual constriction of liquid regions, jointly suppresses further nucleation and growth of secondary α_2_-Al. Some smaller secondary α_2_-Al phases are consumed by larger ones, demonstrating a growth mechanism characteristic of Ostwald ripening.

For the Al-4Si alloy (Fs = 45%), the solute concentration in the residual liquid is approximately 6.61 wt.% Si at the moment of the cooling rate transition (Figure 9). In the early stage of solidification, numerous spherical secondary α_2_-Al nuclei are uniformly distributed in the liquid. Since the solid fraction achieved in Stage I (45%) is well below the equilibrium value at the eutectic temperature (~72%), a substantial amount of liquid remains, resulting in a relatively wide solidification interval during Stage II. The growth of primary α_1_-Al is limited, and secondary α_2_-Al becomes the dominant solidifying phase. As solidification progresses, adjacent secondary α_2_-Al phases undergo coalescence driven by competitive growth, gradually evolving into short-rod morphology.

In the Al-7Si alloy (Fs = 45%), the liquid solute concentration exceeds 11.5 wt.% Si after the cooling rate increase, approaching the eutectic composition (Figure 10). Under such a high solute concentration, the growth of secondary α_2_-Al driven by undercooling is markedly suppressed. As the system rapidly enters the temperature range of the eutectic reaction, these embryos cannot sustain further growth.

## 5. Discussion

### 5.1. Solute Enrichment in the Solid Phase: Synergistic Effect of Cooling Rate and Liquid Solute Concentration

**Figure 11:**
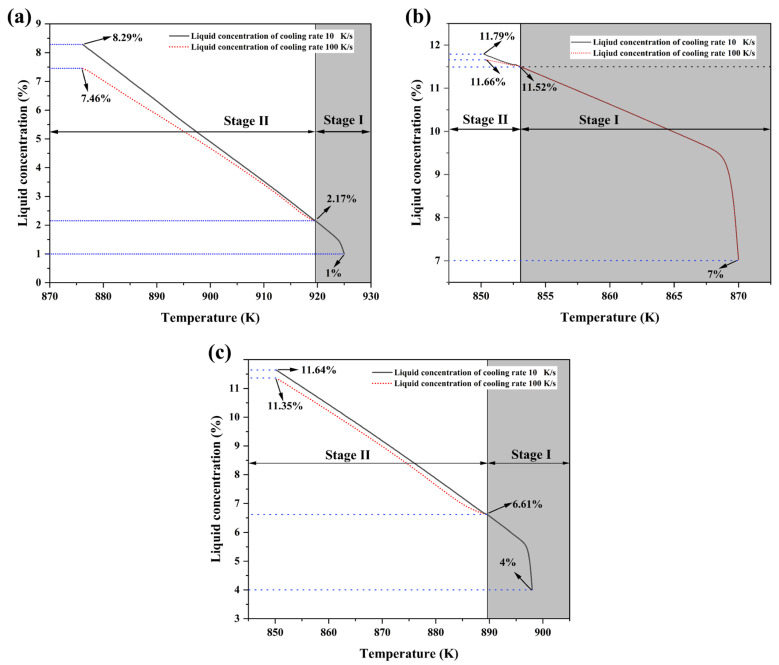
Phase-field simulations reveal the dynamic evolution of solute fields during the continuous cooling process: (**a**) Al-1Si alloy; (**b**) Al-4Si alloy; (**c**) Al-7Si alloy.

To quantitatively trace the solute evolution during secondary solidification, Figure 11 presents the variation of liquid solute concentration with temperature obtained from phase-field simulations under the cooling rates of 100 K/s and 10 K/s. The liquid solute concentration represents the average solute content (in mass percent) within the entire remaining liquid at each solidification moment during continuous cooling, calculated strictly following the principle of mass conservation. Specifically, during the simulation, the local solute field is explicitly resolved and exported; at each output time step, the total solute content in the current liquid region is summed and divided by the liquid fraction at that moment, thereby obtaining the corresponding average liquid concentration. This method ensures a physically consistent and reliable characterization of how the overall liquid composition changes with temperature/solid fraction, thereby providing a direct basis for the subsequent analysis of differences in solute enrichment behavior under different cooling conditions. The simulated curves delineate the complete evolution of solute concentration in the residual liquid for all three alloys, covering the entire thermal evolution from Stage I slurry preparation through the cooling rate switch into Stage II solidification.

The coupling mechanism between solidification kinetics and solute redistribution lies at the core of this synergy. Within the two cooling regimes studied (10 K/s and 100 K/s), the cooling rate of 100 K/s exerts its influence through two pathways: First, it drives a rapid temperature drop in the system, generating significant thermal undercooling that provides the initial driving force for nucleation. Second, based on the Aziz continuous growth model [35,36], the higher cooling rate of 100 K/s suppresses solute diffusion, leading to incomplete solute trapping at the solid/liquid interface, thereby intensifying solute enrichment at the solid front and causing the concentration of the residual liquid to continuously deviate from equilibrium. In contrast, under the cooling rate of 10 K/s, there is sufficient time for solute diffusion, resulting in a weaker degree of solute enrichment. The phase-field results in Figure 10a–c visually demonstrate that at any identical temperature, the liquid concentration under 100 K/s is consistently lower than that under 10 K/s. The concentration data at the final moment of solidification (solidus/eutectic temperature)-Al-1Si (7.46% vs. 8.29%), Al-4Si (11.35% vs. 11.64%), and Al-7Si (11.66% vs. 11.79%) quantitatively confirm that under the conditions studied, the higher cooling rate intensifies solute enrichment.

However, the specific manifestation of solute enrichment is fundamentally governed by the initial alloy composition, which dictates the solute concentration in the residual liquid (*C*_0_). This leads to three distinct solidification paths and nucleation behaviors within the three Al-Si compositions investigated:

For Al-1Si alloy, its low silicon content results in a very low initial solute concentration (*C*_0_ = 2.17%) in the Stage I residual liquid, although the solid fraction has reached 60% in Stage I. The wide solidification interval creates conditions for the combined action of thermal undercooling and constitutional undercooling. As shown in Figure 11a, during the secondary solidification process, the liquid solute concentration difference under 100 K/s and 10 K/s continued to expand as temperature decreased, ultimately reaching a difference of 0.83% (8.29%-10 K/s vs. 7.46%-100 K/s). This indicates that with a lower initial concentration in the system, the suppression of solute diffusion by the cooling rate persists as solidification progresses, driving the explosive nucleation and growth of secondary α_2_-Al.

For the Al-4Si alloy, its intermediate composition leads to an initial concentration (6.61%) that lies between the two extremes and presents typical transitional characteristics. In the initial stage of the secondary solidification process, the high cooling rate significantly enhanced the difference in liquid concentration, demonstrating a strong enrichment effect. However, in the later stage, the concentration difference between 100 K/s and 10 K/s did not continuously expand, as seen in Figure 11b. As solidification proceeded below the solidus temperature, the difference in residual liquid solute concentration converged to 0.29% (11.35%-100 K/s vs. 11.64%-10 K/s). This reflects that under medium liquid concentration, the competition between solute diffusion and solute enrichment is more complex: the higher initial concentration causes the system’s constitutional undercooling to diminish rapidly as the liquid concentration rises, resulting in a relative weakening of the driving effect of solute enrichment.

For the Al-7Si alloy, the residual liquid concentration reached 11.52 wt.% after Stage I. This results in a narrowed solidification interval and significant suppression of the overall undercooling. Nevertheless, Figure 11c shows that the final liquid concentration under 100 K/s (11.66%) remains lower than that under 10 K/s (11.79%), with a concentration difference of 0.13%. This confirms that even under the high-concentration conditions studied, the solute enrichment effect triggered by high cooling rate still exists, providing the necessary local constitutional undercooling driving force for the nucleation of a small number of secondary α_2_-Al phases at the remaining liquid region [37].

These systematic differences in initial solute concentration, stemming directly from the bulk alloy composition, establish the baseline from which the subsequent solute enrichment and its response to cooling rate evolve. By comparing the liquid solute concentration difference between high and medium cooling rates at the end of secondary solidification (Al-1Si: 0.83 wt.%; Al-4Si: 0.29 wt.%; Al-7Si: 0.13 wt.%), it is concluded that within the studied Al-Si system and under the two specific cooling rates, the lower initial liquid solute concentration leads to a more pronounced enhancement of solute enrichment under 100 K/s. Additionally, based on Aziz’s continuous growth model theory and the results established under fixed alloy conditions in this study, a higher cooling rate enhances solute enrichment by suppressing diffusion and promoting solute trapping, and this effect varies monotonically with cooling rate. Based on the trends observed between 10 K/s and 100 K/s, we qualitatively infer that for intermediate cooling rates between 10 K/s and 100 K/s, key metrics such as the extent of solute enrichment, the final liquid concentration difference, and the nucleation density of secondary α_2_-Al are expected to exhibit a continuous transition between the two extreme conditions in each specific alloy we studied. This synergistic effect directly determines the nucleation rate and microstructure morphology of secondary α_2_-Al by regulating the distribution of constitutional undercooling during solidification. These results clarify that, within the scope of this study, high cooling rate and low initial liquid solute concentration jointly affect the secondary solidification process by regulating solid-phase solute enrichment.

### 5.2. Synergistic Regulation of Total Undercooling Contribution and Secondary Solidification Undercooling Mechanism by Solute Concentration and Cooling Rate

The nucleation and growth mechanisms of secondary α_2_-Al within the residual liquid constitute the core focus of this study. According to classical nucleation theory and Equation (4), the nucleation rate J is governed by the nucleation barrier $\Delta G^{*}$, where $\Delta G^{*}\;\propto \;1/\Delta T_{eff}^{2}$ [38,39]. This relationship implies that the nucleation barrier decreases precipitously with the square of the effective undercooling. During Stage II of rheo-diecasting solidification, the effective undercooling $\Delta T_{eff}$ driving the nucleation of secondary α_2_-Al arises from the coupled contributions of thermal and constitutional undercooling [40,41], which can be expressed as:

(9) $$\Delta T_{eff}(t)=\;\{T_{L}[C_{L}^{*}\left(t)\right]-T[C_{L}^{*}\left(t\right)]\}+\{T_{L}(C_{0})-T_{L}[C_{L}^{*}\left(t)]\right\}$$

where $T_{L}(C_{0})$ is the equilibrium liquidus temperature corresponding to the initial average liquid concentration $C_{0}$, $C_{L}^{*}(t)$ is the local liquid concentration at the solid/liquid interface, $T[C_{L}^{*}(t)]$ represents the actual instantaneous temperature, and *T_L_*[$C_{L}^{*}$(*t*)] is the no-equilibrium liquidus temperature corresponding to the local liquid concentration $C_{L}^{*}(t)$. The first term in the equation represents thermal undercooling, while the second term represents constitutional undercooling. During solidification, continuous solute enrichment elevates the liquid concentration at the interface (i.e., $C_{L}^{*}\left(t\right)\;>{\;C}_{0}$). This causes the local liquidus temperature $T_{L}[C_{L}^{*}(t)]$ to drop below the equilibrium liquidus temperature of the liquid $T_{L}(C_{0})$, thereby generating or enhancing constitutional undercooling. Based on classical solidification theory, the constitutional undercooling can be further expressed as [8,41]:

(10) $$\Delta T_{c}=m(c_{L}^{*}-c_{0})$$

where m is the liquidus slope, $c_{L}^{*}$ is the solute concentration on the liquid side of the interface, and $c_{0}$ is the initial solute concentration in the liquid far from the interface. For the Al-Si binary system, the liquidus can be approximated as a linear function based on the equilibrium phase diagram. However, m varies with composition and temperature.

**Figure 12:**
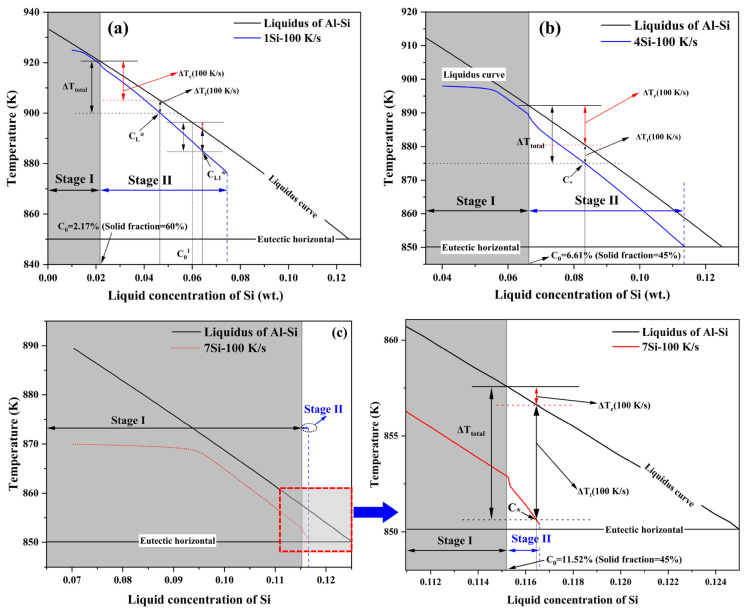
Evolution of liquid solute concentration with temperature and the corresponding undercooling contributions during the secondary solidification of different Al-Si alloys under a high cooling rate (100 K/s): (**a**) Al-1Si alloy; (**b**) Al-4Si alloy; (**c**) Al-7Si alloy.

To elucidate the characteristic contributions of the two undercooling mechanisms in the different alloys, Figure 12 presents the evolution of liquid solute concentration versus temperature obtained from phase-field simulations under a high cooling rate (100 K/s). The relative contributions of thermal and constitutional undercooling are decomposed accordingly. In this work, the instantaneous average liquid solute concentration in the liquid phase derived from the simulation is defined as $c_{L}^{*}$. The initial liquid concentrations *C*_0_ at the onset of the second cooling stage for the respective alloys are: 2.17 wt.% for Al-1Si ($F_{s}\;=\;60\%$), 6.61 wt.% for Al-4Si ($F_{s}\;=\;45\%$), and 11.52 wt.% for Al-7Si ($F_{s}\;=\;45\%$). Because of the solute redistribution effect, the average liquid solute concentration is increasing, narrowing the solidification interval during solidification. This consequently alters the total undercooling, driving a dynamic evolution in the relative undercooling contributions. Based on the simulated solute and temperature fields at 100 K/s, the following analysis for the three studied Al-Si alloys provides an interpretive perspective on how their undercooling contributions are governed by their respective initial compositions and evolving solute concentrations:

As shown in Figure 12, a comparative analysis of the three specific alloys—Al-1Si, Al-4Si, and Al-7Si—reveals that the nucleation driving force for secondary α_2_-Al is governed by both constitutional and thermal undercooling. However, their relative strength varies systematically with alloy composition and evolves dynamically during solidification. A comparative analysis across the alloys reveals a clear spectrum of behavior:

A key difference is evident at the initial stage of secondary solidification, which shows a clear difference in undercooling contribution between Al-1Si and Al-7Si. For the Al-1Si alloy (Figure 12a), with a low initial solute concentration and a wide solidification interval, the slope of the solidification curve (derived from the phase diagram in Figure 1) is relatively steep. This implies that fluctuations in interfacial solute concentration can significantly change the local liquidus temperature. Consequently, during the initial stage of secondary solidification, local composition changes caused by solute redistribution effectively induce constitutional undercooling. Based on the comparative analysis between Al-1Si (Figure 12a) and Al-7Si (Figure 12c), constitutional undercooling makes a more substantial contribution to the effective undercooling required for nucleation of secondary α_2_-Al in Al-1Si than in Al-7Si alloy during this early stage. In contrast, for the Al-7Si alloy (Figure 12c), the initial concentration is already close to the eutectic composition (~12.6 wt.%), resulting in an extremely narrow solidification temperature range and a significantly reduced total undercooling. Under these conditions, the impact of minor compositional variations on the liquidus temperature is weakened. Consequently, the driving force for nucleation is derived primarily from thermal undercooling throughout the process. For the Al-4Si alloy (Figure 12b), with the intermediate initial solute concentration, the undercooling behavior exhibits a distinct behavior in the initial stage, where thermal and constitutional undercooling act in synergy, jointly contributing to the nucleation driving force.

As solidification proceeds, the evolution of the undercooling contributions follows a consistent trend across all three alloys. The continuous solute enrichment of the residual liquid (Figure 11) systematically narrows the solidification interval. This leads to a continuous decrease in the total system undercooling and also affects the subsequent undercooling contribution. The relative role of thermal undercooling is enhanced in providing the driving force for continued nucleation. Consequently, while thermal undercooling remains the primary contributor throughout in Al-7Si, the system in Al-1Si undergoes a dynamic shift in the primary source of undercooling from a stronger reliance on constitutional effects toward a greater influence of thermal undercooling in later stages. In Al-4Si, this progression means that the initially synergistic contribution evolves toward a state where thermal undercooling becomes progressively more dominant.

Under the cooling rate of 10 K/s, although the overall magnitude of undercooling for the system decreases, the comparative trends in the relative contributions of thermal and constitutional undercooling remain consistent with those observed at 100 K/s. This indicates that the cooling rate primarily regulates the absolute magnitude of undercooling and the degree of solute trapping, without altering the comparative sequence or the dynamic evolution of the relative undercooling contributions, which are determined by initial solute concentration and the solidification range of Al-Si alloy.

In summary, the nucleation and evolution of secondary α_2_-Al in the studied Al-Si alloys under high-solid-fraction rheo-diecasting conditions is synergistically governed by thermal undercooling and constitutional undercooling, with their relative importance evolving dynamically according to the initial solute concentration of stage II and the progress of solidification. The reason for this evolution lies in a “thermo-solute coupling effect” driven by the interaction between “solute enrichment” and “narrowing of the solidification interval”. At the early stage of solidification, constitutional undercooling gives rise to solute enrichment in secondary α_2_-Al. As solidification proceeds, the overall solute concentration in the liquid increases, leading to a systematic contraction of the solidification interval, thereby enhancing the role of thermal undercooling. In the Al-7Si alloy, as the initial state is already close to the eutectic composition, thermal undercooling remains the primary contributor throughout the entire process. This dynamic driving force is established from the perspective of comparing undercooling contributions across the three alloy compositions. Thus, it provides a core theoretical framework for a profound understanding of the nucleation and evolution differences of secondary α_2_-Al during secondary solidification in Al-Si alloys (1–7 wt.%) under high-solid-fraction rheo-diecasting conditions. Additionally, as a speculative extension beyond the current study, for hypoeutectic alloys with high solid solubility (e.g., Al-Cu, Al-Mg): Their broad primary solidification range and high solute supersaturation may lead to secondary solidification behavior similar to that of Al-1Si in this study, where constitutional undercooling likely plays a key role in the early stages. However, the dynamics of solute enrichment and the final microstructural evolution would be different.

## 6. Conclusions

This study systematically elucidates the synergistic effect of cooling rate and solute concentration on governing the nucleation and growth of secondary α_2_-Al during high-solid-fraction rheo-diecasting of Al-xSi (x = 1, 4, 7 wt.%) alloys, via integrated gradient-cooling experiments (100 vs. 10 K/s) and multiscale phase-field simulations. The key findings and interpretations are strictly constrained within the studied range of compositions (1–7 wt.% Si) and the two specific cooling rates (10 and 100 K/s), and are summarized as follows:

(1) Both experiments and simulations confirm that the nucleation density and morphology of secondary α_2_-Al are predominantly co-controlled by the initial Si content and the applied cooling rate within the studied range. A lower initial Si concentration significantly enhances the effect of cooling rate on solute enrichment. In Al-1Si alloy, higher cooling (100 K/s) promotes fine, uniformly distributed particles, while lower cooling (10 K/s) leads to coarsening and coalescence. While the Al-4Si alloy shows a transitional behavior with abundant secondary α_2_-Al that readily coalesces at a lower cooling rate. However, the Al-7Si alloy, with its near-eutectic residual liquid, exhibits strongly suppressed nucleation under both cooling rates studied.
(2) The secondary solidification in the studied Al-Si alloys under the applied cooling conditions is governed by a coupling between cooling rate and initial liquid solute concentration. High cooling rate of 100 K/s promotes solute enrichment ahead of the solidification front by restricting solute diffusion and giving rise to incomplete solute trapping. This effect is strongly coupled with the alloy initial solute content: the lower the initial concentration, the more strongly the solute enrichment is amplified under a high cooling rate, as evidenced by the final liquid concentration difference between 100 K/s and 10 K/s conditions: Al-1Si (0.83 wt.%) > Al-4Si (0.29 wt.%) > Al-7Si (0.13 wt.%).
(3) A comparative study of the three specific alloys (Al-1Si, Al-4Si, and Al-7Si) under high-solid-fraction rheo-diecasting conditions reveals that the nucleation of secondary α_2_-Al is governed by a dynamic competition between thermal and constitutional undercooling. The relative importance varies with the evolving liquid solute concentration and solidification pathway of each alloy. Specifically, constitutional undercooling contributes a substantially greater driving force for nucleation in Al-1Si compared to Al-7Si in early stage II. Meanwhile, in Al-7Si, the near-eutectic residual liquid narrows the solidification interval, making thermal undercooling the primary driver.

## Figures and Tables

**Table 1:** Chemical composition of Al-xSi alloy used in this study (wt.%).

Alloy	Si	Fe	Al
Al-1Si	1.18	0.076	Bal.
Al-4Si	4.25	0.088	Bal.
Al-7Si	7.32	0.096	Bal.

**Table 2:** Thermodynamic and kinetic parameters of Al-xSi alloy [19].

Definition	Symbol	Value [Unit]
Phase-field mobility	*M_Φ_*	0.34 [m^3^·J^−1^s^−1^]
Anisotropy strength	*ε*	0.01
Initial solute concentration	*C*_0_	1/4/7 [mass%]
Diffusion coefficient for Si in liquid	*D_L_*	2 × 10^−9^ [m^2^·s^−1^]
Diffusion coefficient for Si in solid	*D_S_*	1 × 10^−13^ [m^2^·s^−1^]
Coupling constant	*λ*	5.0
Capillary length	*d*	0.2 [μm]
Solute partition coefficient	*k*	CALPHAD calculated
Liquidus	*T_Liquidus_*	CALPHAD calculated
Eutectic/Solidus temperature	*T_eutectic_*	CALPHAD calculated
Melting point of pure solvent	*T_M_*	CALPHAD calculated
Nucleation parameter	*k*_1_	5 × 10^−2^
Nucleation parameter	*k*_2_	240

## Data Availability

The original contributions presented in this study are included in the article. Further inquiries can be directed to the corresponding authors.
